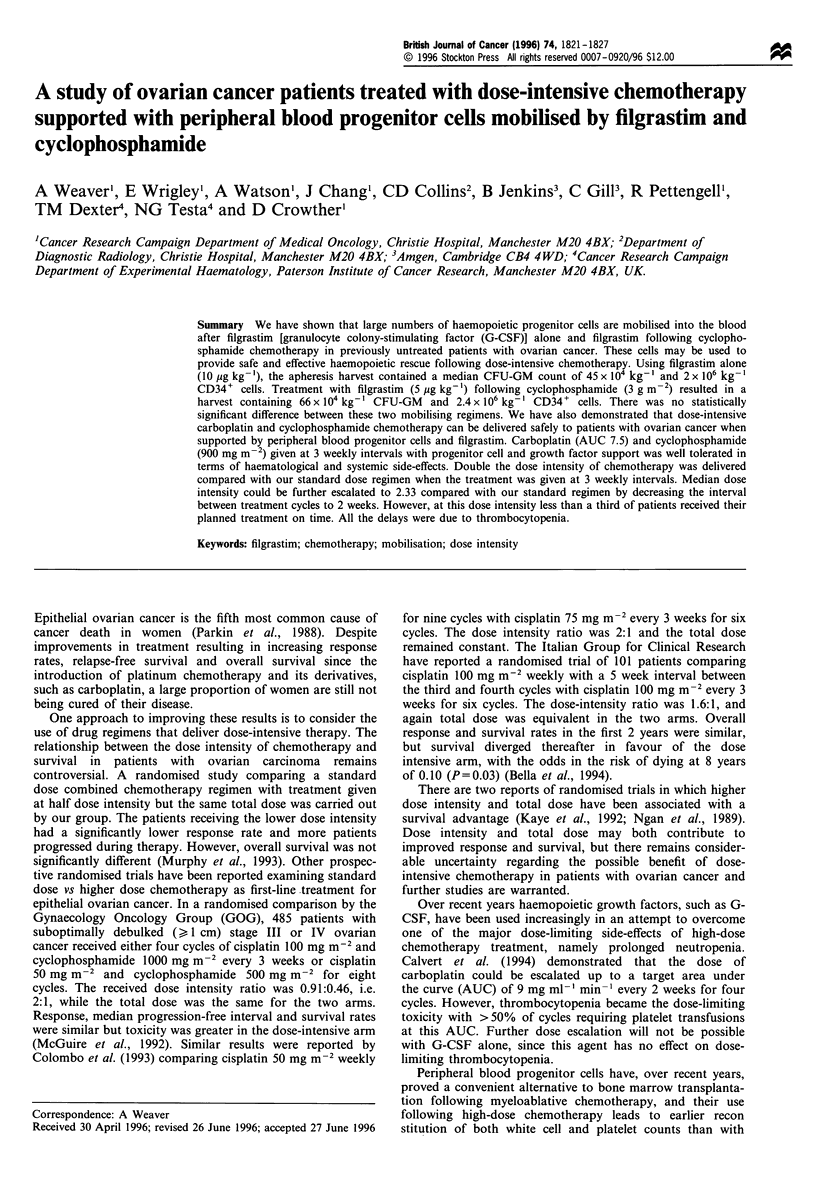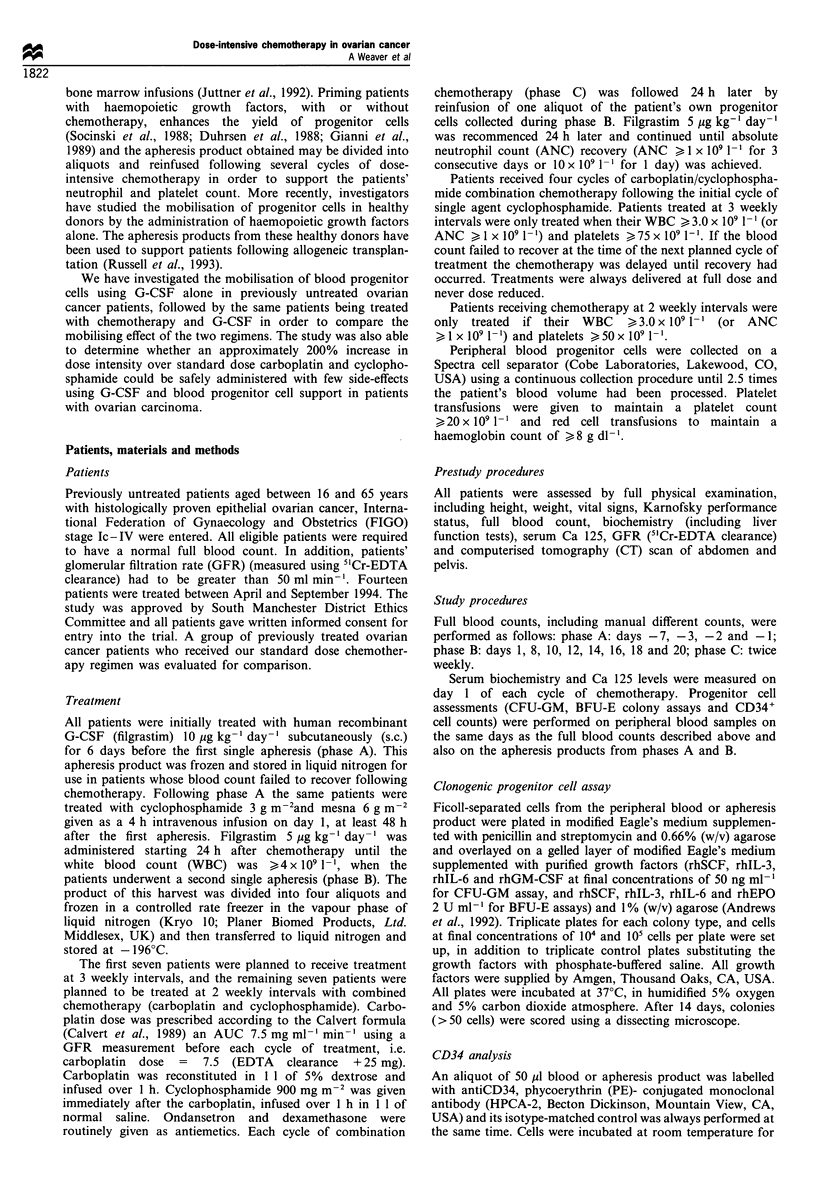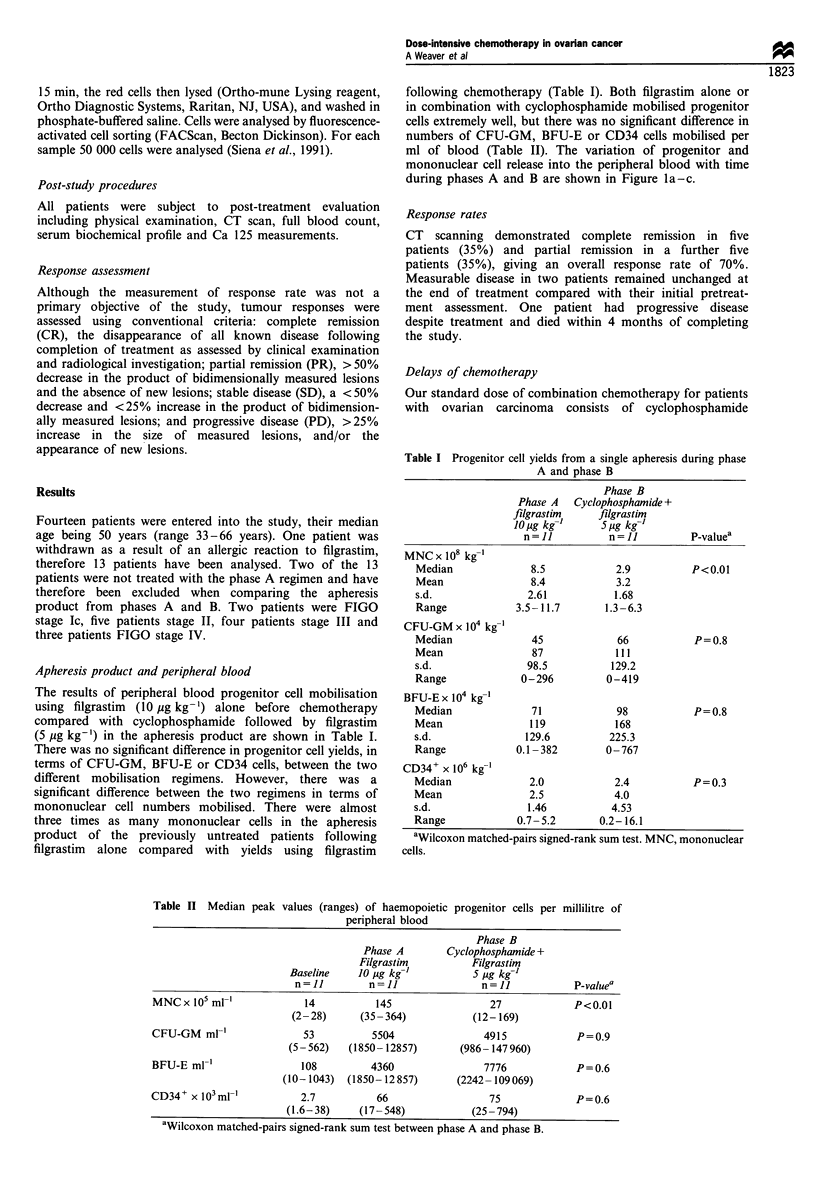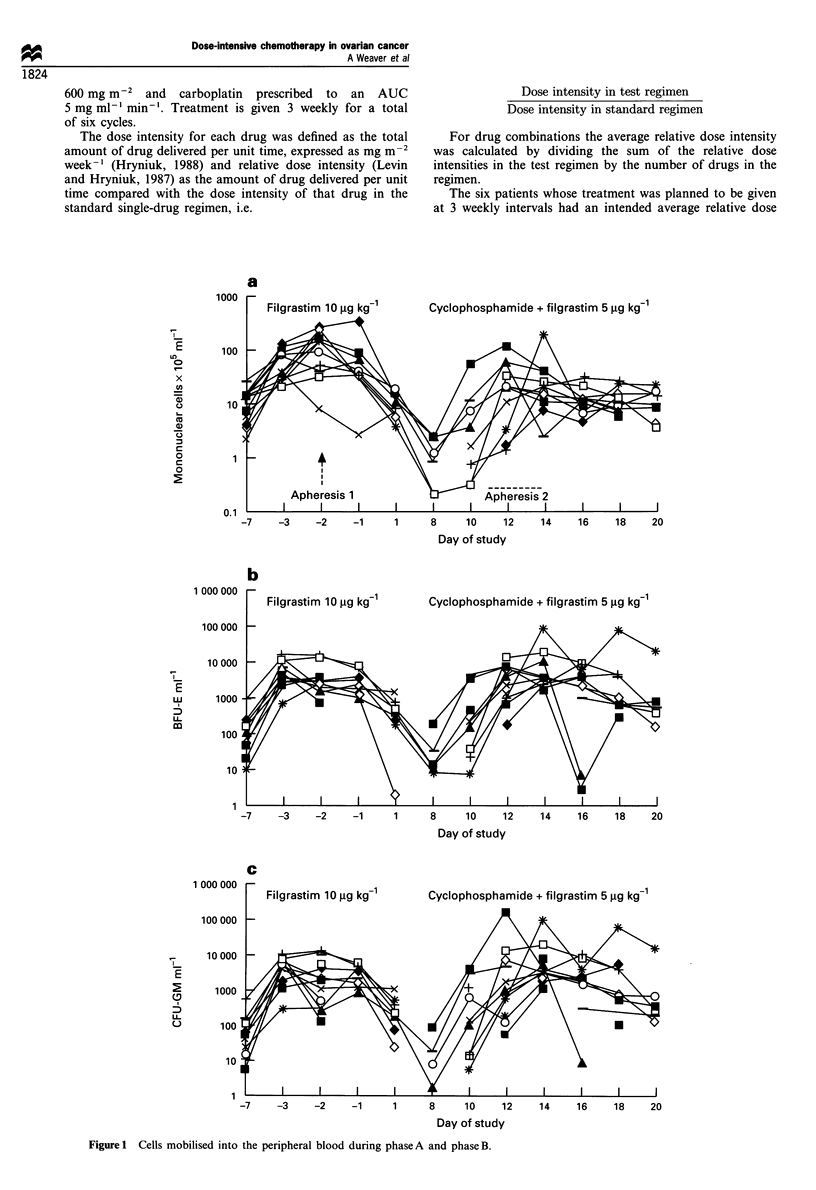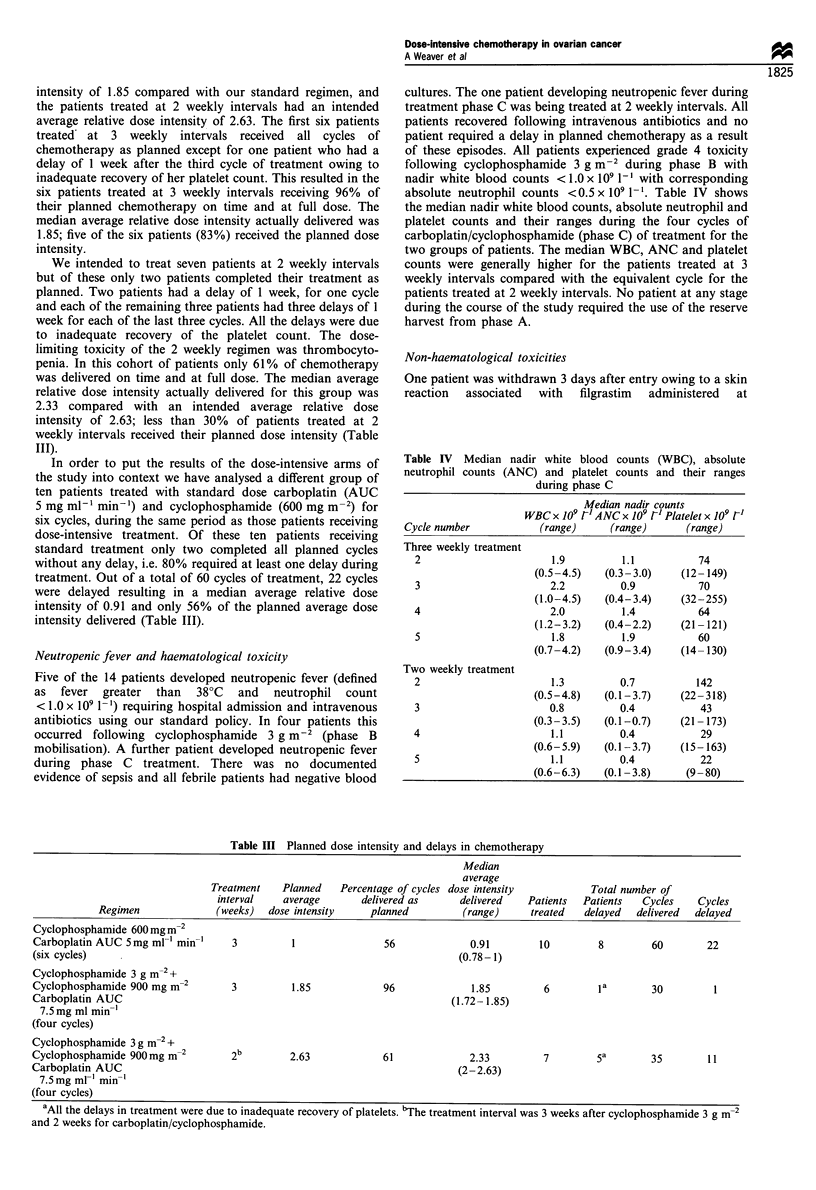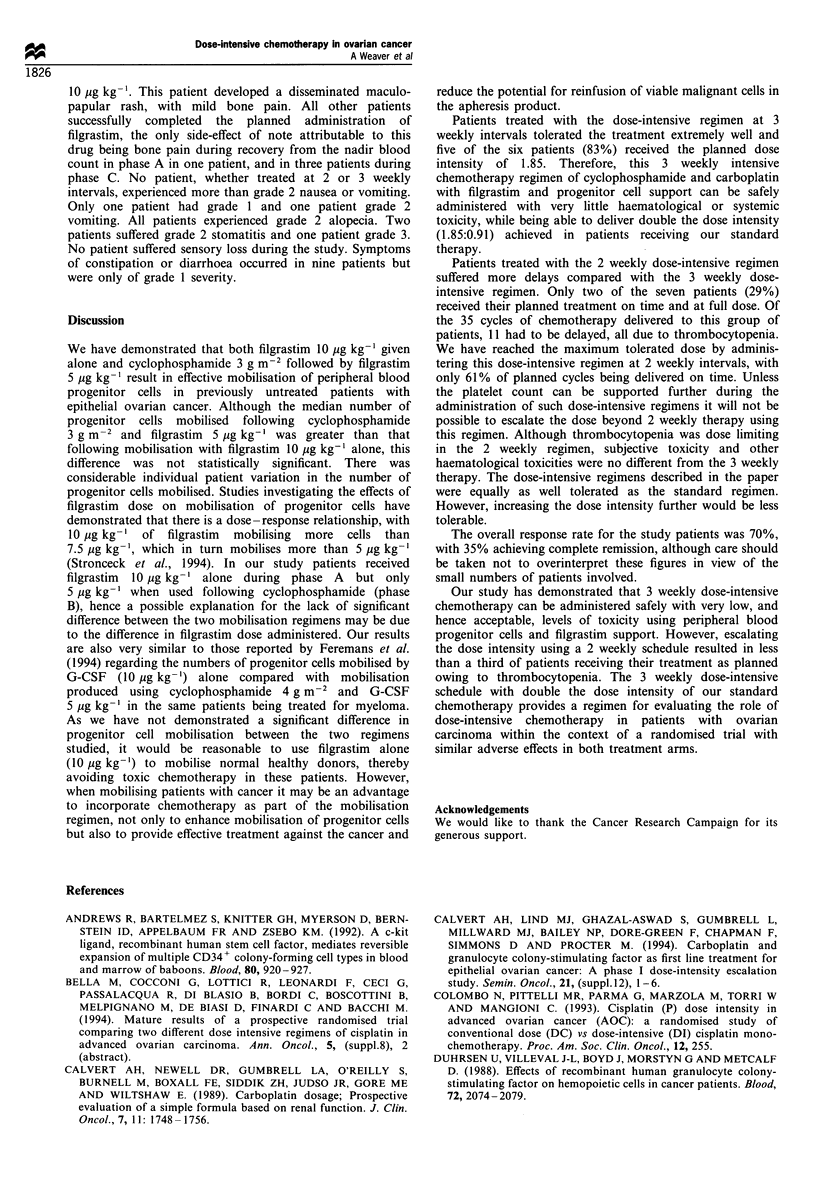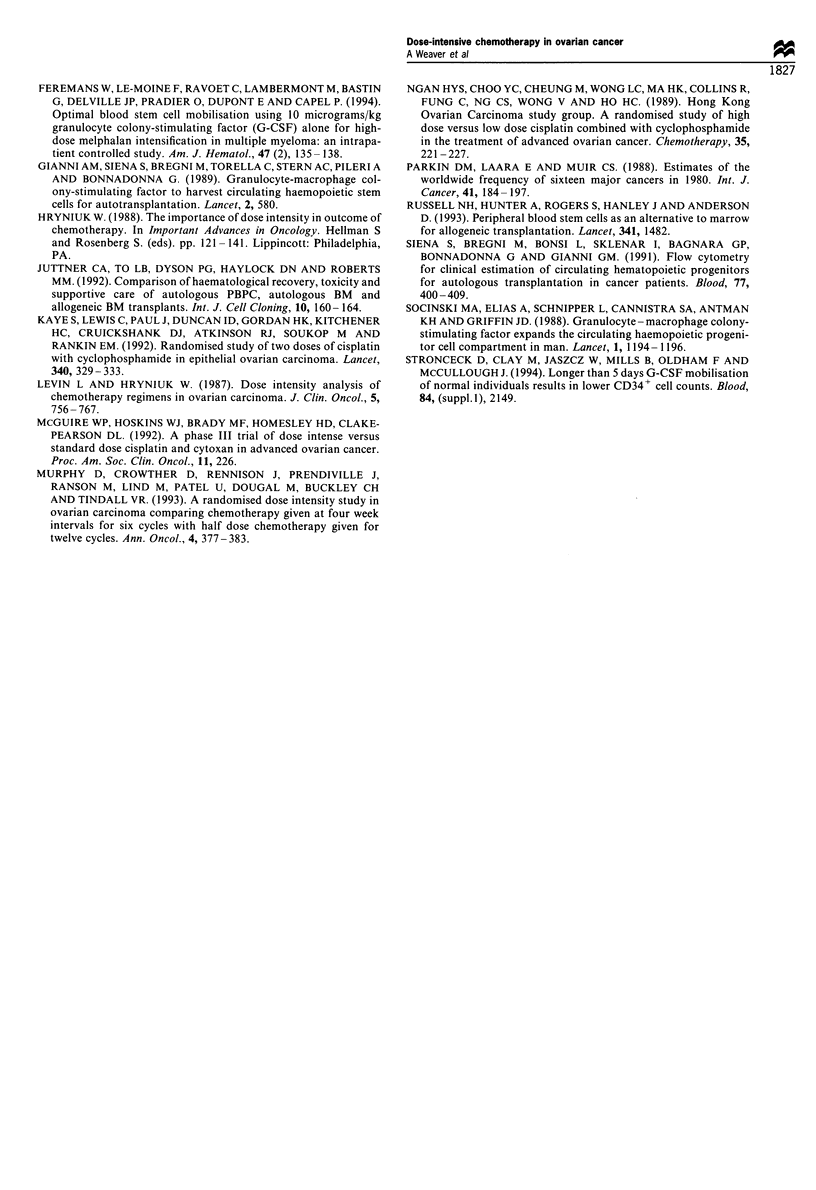# A study of ovarian cancer patients treated with dose-intensive chemotherapy supported with peripheral blood progenitor cells mobilised by filgrastim and cyclophosphamide.

**DOI:** 10.1038/bjc.1996.637

**Published:** 1996-12

**Authors:** A. Weaver, E. Wrigley, A. Watson, J. Chang, C. D. Collins, B. Jenkins, C. Gill, R. Pettengell, T. M. Dexter, N. G. Testa, D. Crowther

**Affiliations:** Cancer Research Campaign Department of Medical Oncology, Christie Hospital, Manchester, UK.

## Abstract

We have shown that large numbers of haemopoietic progenitor cells are mobilised into the blood after filgrastim [granulocyte colony-stimulating factor (G-CSF)] alone and filgrastim following cyclophosphamide chemotherapy in previously untreated patients with ovarian cancer. These cells may be used to provide safe and effective haemopoietic rescue following dose-intensive chemotherapy. Using filgrastim alone (10 micrograms kg-1), the apheresis harvest contained a median CFU-GM count of 45 x 10(4) kg-1 and 2 x 10(6) kg-1 CD34+ cells. Treatment with filgrastim (5 micrograms kg-1) following cyclophosphamide (3 g m-2) resulted in a harvest containing 66 x 10(4) kg-1 CFU-GM and 2.4 x 10(6) kg-1 CD34+ cells. There was no statistically significant difference between these two mobilising regimens. We have also demonstrated that dose-intensive carboplatin and cyclophosphamide chemotherapy can be delivered safely to patients with ovarian cancer when supported by peripheral blood progenitor cells and filgrastim. Carboplatin (AUC 7.5) and cyclophosphamide (900 mg m-2) given at 3 weekly intervals with progenitor cell and growth factor support was well tolerated in terms of haematological and systemic side-effects. Double the dose intensity of chemotherapy was delivered compared with our standard dose regimen when the treatment was given at 3 weekly intervals. Median dose intensity could be further escalated to 2.33 compared with our standard regimen by decreasing the interval between treatment cycles to 2 weeks. However, at this dose intensity less than a third of patients received their planned treatment on time. All the delays were due to thrombocytopenia.


British Journal of Cancer (1996) 74, 1821-1827
© 1996 Stockton Press All rights reserved 0007-0920/96 $12.00

### A study of ovarian cancer patients treated with dose-intensive chemotherapy supported with peripheral blood progenitor cells mobilised by filgrastim and cyclophosphamide

A Weaver[1], E Wrigley[1], A Watson[1], J Chang[1], CD Collins[2], B Jenkins[3], C Gill[3], R Pettengell[1], TM Dexter[4], NG Testa[4] and D Crowther[1]

[1]*Cancer Research Campaign Department of Medical Oncology, Christie Hospital, Manchester M20 4BX;* [2]*Department of Diagnostic Radiology, Christie Hospital, Manchester M20 4BX;* [3]*Amgen, Cambridge CB4 4WD;* [4]*Cancer Research Campaign Department of Experimental Haematology, Paterson Institute of Cancer Research, Manchester M20 4BX, UK.*

**Summary** We have shown that large numbers of haemopoietic progenitor cells are mobilised into the blood after filgrastim [granulocyte colony-stimulating factor (G-CSF)] alone and filgrastim following cyclophosphamide chemotherapy in previously untreated patients with ovarian cancer. These cells may be used to provide safe and effective haemopoietic rescue following dose-intensive chemotherapy. Using filgrastim alone (10 $\mu g\ kg^{-1}$), the apheresis harvest contained a median CFU-GM count of $45\times10^4\ kg^{-1}$ and $2\times10^6\ kg^{-1}$ $CD34^+$ cells. Treatment with filgrastim (5 $\mu g\ kg^{-1}$) following cyclophosphamide (3 $g\ m^{-2}$) resulted in a harvest containing $66\times10^4\ kg^{-1}$ CFU-GM and $2.4\times10^6\ kg^{-1}$ $CD34^+$ cells. There was no statistically significant difference between these two mobilising regimens. We have also demonstrated that dose-intensive carboplatin and cyclophosphamide chemotherapy can be delivered safely to patients with ovarian cancer when supported by peripheral blood progenitor cells and filgrastim. Carboplatin (AUC 7.5) and cyclophosphamide (900 $mg\ m^{-2}$) given at 3 weekly intervals with progenitor cell and growth factor support was well tolerated in terms of haematological and systemic side-effects. Double the dose intensity of chemotherapy was delivered compared with our standard dose regimen when the treatment was given at 3 weekly intervals. Median dose intensity could be further escalated to 2.33 compared with our standard regimen by decreasing the interval between treatment cycles to 2 weeks. However, at this dose intensity less than a third of patients received their planned treatment on time. All the delays were due to thrombocytopenia.

**Keywords:** filgrastim; chemotherapy; mobilisation; dose intensity

Epithelial ovarian cancer is the fifth most common cause of cancer death in women (Parkin *et al.*, 1988). Despite improvements in treatment resulting in increasing response rates, relapse-free survival and overall survival since the introduction of platinum chemotherapy and its derivatives, such as carboplatin, a large proportion of women are still not being cured of their disease.

One approach to improving these results is to consider the use of drug regimens that deliver dose-intensive therapy. The relationship between the dose intensity of chemotherapy and survival in patients with ovarian carcinoma remains controversial. A randomised study comparing a standard dose combined chemotherapy regimen with treatment given at half dose intensity but the same total dose was carried out by our group. The patients receiving the lower dose intensity had a significantly lower response rate and more patients progressed during therapy. However, overall survival was not significantly different (Murphy *et al.*, 1993). Other prospective randomised trials have been reported examining standard dose *vs* higher dose chemotherapy as first-line treatment for epithelial ovarian cancer. In a randomised comparison by the Gynaecology Oncology Group (GOG), 485 patients with suboptimally debulked ($\geq 1$ cm) stage III or IV ovarian cancer received either four cycles of cisplatin 100 $mg\ m^{-2}$ and cyclophosphamide 1000 $mg\ m^{-2}$ every 3 weeks or cisplatin 50 $mg\ m^{-2}$ and cyclophosphamide 500 $mg\ m^{-2}$ for eight cycles. The received dose intensity ratio was 0.91:0.46, i.e. 2:1, while the total dose was the same for the two arms. Response, median progression-free interval and survival rates were similar but toxicity was greater in the dose-intensive arm (McGuire *et al.*, 1992). Similar results were reported by Colombo *et al.* (1993) comparing cisplatin 50 $mg\ m^{-2}$ weekly for nine cycles with cisplatin 75 $mg\ m^{-2}$ every 3 weeks for six cycles. The dose intensity ratio was 2:1 and the total dose remained constant. The Italian Group for Clinical Research have reported a randomised trial of 101 patients comparing cisplatin 100 $mg\ m^{-2}$ weekly with a 5 week interval between the third and fourth cycles with cisplatin 100 $mg\ m^{-2}$ every 3 weeks for six cycles. The dose-intensity ratio was 1.6:1, and again total dose was equivalent in the two arms. Overall response and survival rates in the first 2 years were similar, but survival diverged thereafter in favour of the dose intensive arm, with the odds in the risk of dying at 8 years of 0.10 ($P=0.03$) (Bella *et al.*, 1994).

There are two reports of randomised trials in which higher dose intensity and total dose have been associated with a survival advantage (Kaye *et al.*, 1992; Ngan *et al.*, 1989). Dose intensity and total dose may both contribute to improved response and survival, but there remains considerable uncertainty regarding the possible benefit of dose-intensive chemotherapy in patients with ovarian cancer and further studies are warranted.

Over recent years haemopoietic growth factors, such as G-CSF, have been used increasingly in an attempt to overcome one of the major dose-limiting side-effects of high-dose chemotherapy treatment, namely prolonged neutropenia. Calvert *et al.* (1994) demonstrated that the dose of carboplatin could be escalated up to a target area under the curve (AUC) of 9 $mg\ ml^{-1}\ min^{-1}$ every 2 weeks for four cycles. However, thrombocytopenia became the dose-limiting toxicity with >50% of cycles requiring platelet transfusions at this AUC. Further dose escalation will not be possible with G-CSF alone, since this agent has no effect on dose-limiting thrombocytopenia.

Peripheral blood progenitor cells have, over recent years, proved a convenient alternative to bone marrow transplantation following myeloablative chemotherapy, and their use following high-dose chemotherapy leads to earlier recon stitution of both white cell and platelet counts than with

Correspondence: A Weaver
Received 30 April 1996; revised 26 June 1996; accepted 27 June 1996

bone marrow infusions (Juttner et al., 1992). Priming patients with haemopoietic growth factors, with or without chemotherapy, enhances the yield of progenitor cells (Socinski et al., 1988; Duhrsen et al., 1988; Gianni et al., 1989) and the apheresis product obtained may be divided into aliquots and reinfused following several cycles of dose-intensive chemotherapy in order to support the patients' neutrophil and platelet count. More recently, investigators have studied the mobilisation of progenitor cells in healthy donors by the administration of haemopoietic growth factors alone. The apheresis products from these healthy donors have been used to support patients following allogeneic transplantation (Russell et al., 1993).

We have investigated the mobilisation of blood progenitor cells using G-CSF alone in previously untreated ovarian cancer patients, followed by the same patients being treated with chemotherapy and G-CSF in order to compare the mobilising effect of the two regimens. The study was also able to determine whether an approximately 200% increase in dose intensity over standard dose carboplatin and cyclophosphamide could be safely administered with few side-effects using G-CSF and blood progenitor cell support in patients with ovarian carcinoma.

### Patients, materials and methods

### *Patients*

Previously untreated patients aged between 16 and 65 years with histologically proven epithelial ovarian cancer, International Federation of Gynaecology and Obstetrics (FIGO) stage Ic–IV were entered. All eligible patients were required to have a normal full blood count. In addition, patients' glomerular filtration rate (GFR) (measured using $^{51}$Cr-EDTA clearance) had to be greater than 50 ml min$^{-1}$. Fourteen patients were treated between April and September 1994. The study was approved by South Manchester District Ethics Committee and all patients gave written informed consent for entry into the trial. A group of previously treated ovarian cancer patients who received our standard dose chemotherapy regimen was evaluated for comparison.

### *Treatment*

All patients were initially treated with human recombinant G-CSF (filgrastim) 10 $\mu$g kg$^{-1}$ day$^{-1}$ subcutaneously (s.c.) for 6 days before the first single apheresis (phase A). This apheresis product was frozen and stored in liquid nitrogen for use in patients whose blood count failed to recover following chemotherapy. Following phase A the same patients were treated with cyclophosphamide 3 g m$^{-2}$and mesna 6 g m$^{-2}$ given as a 4 h intravenous infusion on day 1, at least 48 h after the first apheresis. Filgrastim 5 $\mu$g kg$^{-1}$ day$^{-1}$ was administered starting 24 h after chemotherapy until the white blood count (WBC) was $\geqslant 4 \times 10^9$ l$^{-1}$, when the patients underwent a second single apheresis (phase B). The product of this harvest was divided into four aliquots and frozen in a controlled rate freezer in the vapour phase of liquid nitrogen (Kryo 10; Planer Biomed Products, *Ltd.* Middlesex, UK) and then transferred to liquid nitrogen and stored at −196°C.

The first seven patients were planned to receive treatment at 3 weekly intervals, and the remaining seven patients were planned to be treated at 2 weekly intervals with combined chemotherapy (carboplatin and cyclophosphamide). Carboplatin dose was prescribed according to the Calvert formula (Calvert et al., 1989) an AUC 7.5 mg ml$^{-1}$ min$^{-1}$ using a GFR measurement before each cycle of treatment, i.e. carboplatin dose = 7.5 (EDTA clearance +25 mg). Carboplatin was reconstituted in 1 l of 5% dextrose and infused over 1 h. Cyclophosphamide 900 mg m$^{-2}$ was given immediately after the carboplatin, infused over 1 h in 1 l of normal saline. Ondansetron and dexamethasone were routinely given as antiemetics. Each cycle of combination chemotherapy (phase C) was followed 24 h later by reinfusion of one aliquot of the patient's own progenitor cells collected during phase B. Filgrastim 5 $\mu$g kg$^{-1}$ day$^{-1}$ was recommenced 24 h later and continued until absolute neutrophil count (ANC) recovery (ANC $\geqslant 1 \times 10^9$ l$^{-1}$ for 3 consecutive days or $10 \times 10^9$ l$^{-1}$ for 1 day) was achieved.

Patients received four cycles of carboplatin/cyclophosphamide combination chemotherapy following the initial cycle of single agent cyclophosphamide. Patients treated at 3 weekly intervals were only treated when their WBC $\geqslant 3.0 \times 10^9$ l$^{-1}$ (or ANC $\geqslant 1 \times 10^9$ l$^{-1}$) and platelets $\geqslant 75 \times 10^9$ l$^{-1}$. If the blood count failed to recover at the time of the next planned cycle of treatment the chemotherapy was delayed until recovery had occurred. Treatments were always delivered at full dose and never dose reduced.

Patients receiving chemotherapy at 2 weekly intervals were only treated if their WBC $\geqslant 3.0 \times 10^9$ l$^{-1}$ (or ANC $\geqslant 1 \times 10^9$ l$^{-1}$) and platelets $\geqslant 50 \times 10^9$ l$^{-1}$.

Peripheral blood progenitor cells were collected on a Spectra cell separator (Cobe Laboratories, Lakewood, CO, USA) using a continuous collection procedure until 2.5 times the patient's blood volume had been processed. Platelet transfusions were given to maintain a platelet count $\geqslant 20 \times 10^9$ l$^{-1}$ and red cell transfusions to maintain a haemoglobin count of $\geqslant 8$ g dl$^{-1}$.

### *Prestudy procedures*

All patients were assessed by full physical examination, including height, weight, vital signs, Karnofsky performance status, full blood count, biochemistry (including liver function tests), serum Ca 125, GFR ($^{51}$Cr-EDTA clearance) and computerised tomography (CT) scan of abdomen and pelvis.

### *Study procedures*

Full blood counts, including manual different counts, were performed as follows: phase A: days −7, −3, −2 and −1; phase B: days 1, 8, 10, 12, 14, 16, 18 and 20; phase C: twice weekly.

Serum biochemistry and Ca 125 levels were measured on day 1 of each cycle of chemotherapy. Progenitor cell assessments (CFU-GM, BFU-E colony assays and CD34$^+$ cell counts) were performed on peripheral blood samples on the same days as the full blood counts described above and also on the apheresis products from phases A and B.

### *Clonogenic progenitor cell assay*

Ficoll-separated cells from the peripheral blood or apheresis product were plated in modified Eagle's medium supplemented with penicillin and streptomycin and 0.66% (w/v) agarose and overlayed on a gelled layer of modified Eagle's medium supplemented with purified growth factors (rhSCF, rhIL-3, rhIL-6 and rhGM-CSF at final concentrations of 50 ng ml$^{-1}$ for CFU-GM assay, and rhSCF, rhIL-3, rhIL-6 and rhEPO 2 U ml$^{-1}$ for BFU-E assays) and 1% (w/v) agarose (Andrews et al., 1992). Triplicate plates for each colony type, and cells at final concentrations of $10^4$ and $10^5$ cells per plate were set up, in addition to triplicate control plates substituting the growth factors with phosphate-buffered saline. All growth factors were supplied by Amgen, Thousand Oaks, CA, USA. All plates were incubated at 37°C, in humidified 5% oxygen and 5% carbon dioxide atmosphere. After 14 days, colonies (>50 cells) were scored using a dissecting microscope.

### *CD34 analysis*

An aliquot of 50 $\mu$l blood or apheresis product was labelled with antiCD34, phycoerythrin (PE)- conjugated monoclonal antibody (HPCA-2, Becton Dickinson, Mountain View, CA, USA) and its isotype-matched control was always performed at the same time. Cells were incubated at room temperature for

15 min, the red cells then lysed (Ortho-mune Lysing reagent, Ortho Diagnostic Systems, Raritan, NJ, USA), and washed in phosphate-buffered saline. Cells were analysed by fluorescence-activated cell sorting (FACScan, Becton Dickinson). For each sample 50 000 cells were analysed (Siena *et al*., 1991).

### *Post-study procedures*

All patients were subject to post-treatment evaluation including physical examination, CT scan, full blood count, serum biochemical profile and Ca 125 measurements.

### *Response assessment*

Although the measurement of response rate was not a primary objective of the study, tumour responses were assessed using conventional criteria: complete remission (CR), the disappearance of all known disease following completion of treatment as assessed by clinical examination and radiological investigation; partial remission (PR), >50% decrease in the product of bidimensionally measured lesions and the absence of new lesions; stable disease (SD), a <50% decrease and <25% increase in the product of bidimensionally measured lesions; and progressive disease (PD), >25% increase in the size of measured lesions, and/or the appearance of new lesions.

### Results

Fourteen patients were entered into the study, their median age being 50 years (range 33–66 years). One patient was withdrawn as a result of an allergic reaction to filgrastim, therefore 13 patients have been analysed. Two of the 13 patients were not treated with the phase A regimen and have therefore been excluded when comparing the apheresis product from phases A and B. Two patients were FIGO stage Ic, five patients stage II, four patients stage III and three patients FIGO stage IV.

### *Apheresis product and peripheral blood*

The results of peripheral blood progenitor cell mobilisation using filgrastim (10 $\mu g\ kg^{-1}$) alone before chemotherapy compared with cyclophosphamide followed by filgrastim (5 $\mu g\ kg^{-1}$) in the apheresis product are shown in Table I. There was no significant difference in progenitor cell yields, in terms of CFU-GM, BFU-E or CD34 cells, between the two different mobilisation regimens. However, there was a significant difference between the two regimens in terms of mononuclear cell numbers mobilised. There were almost three times as many mononuclear cells in the apheresis product of the previously untreated patients following filgrastim alone compared with yields using filgrastim following chemotherapy (Table I). Both filgrastim alone or in combination with cyclophosphamide mobilised progenitor cells extremely well, but there was no significant difference in numbers of CFU-GM, BFU-E or CD34 cells mobilised per ml of blood (Table II). The variation of progenitor and mononuclear cell release into the peripheral blood with time during phases A and B are shown in Figure 1a–c.

### *Response rates*

CT scanning demonstrated complete remission in five patients (35%) and partial remission in a further five patients (35%), giving an overall response rate of 70%. Measurable disease in two patients remained unchanged at the end of treatment compared with their initial pretreatment assessment. One patient had progressive disease despite treatment and died within 4 months of completing the study.

### *Delays of chemotherapy*

Our standard dose of combination chemotherapy for patients with ovarian carcinoma consists of cyclophosphamide

**Table I** Progenitor cell yields from a single apheresis during phase A and phase B

| | *Phase A filgrastim 10 $\mu g\ kg^{-1}$* n=*11* | *Phase B Cyclophosphamide + filgrastim 5 $\mu g\ kg^{-1}$* n=*11* | P-value[a] |
|---|---|---|---|
| MNC × $10^8\ kg^{-1}$ | | | |
| Median | 8.5 | 2.9 | $P$<0.01 |
| Mean | 8.4 | 3.2 | |
| s.d. | 2.61 | 1.68 | |
| Range | 3.5–11.7 | 1.3–6.3 | |
| CFU-GM × $10^4\ kg^{-1}$ | | | |
| Median | 45 | 66 | $P$=0.8 |
| Mean | 87 | 111 | |
| s.d. | 98.5 | 129.2 | |
| Range | 0–296 | 0–419 | |
| BFU-E × $10^4\ kg^{-1}$ | | | |
| Median | 71 | 98 | $P$=0.8 |
| Mean | 119 | 168 | |
| s.d. | 129.6 | 225.3 | |
| Range | 0.1–382 | 0–767 | |
| $CD34^+ \times 10^6\ kg^{-1}$ | | | |
| Median | 2.0 | 2.4 | $P$=0.3 |
| Mean | 2.5 | 4.0 | |
| s.d. | 1.46 | 4.53 | |
| Range | 0.7–5.2 | 0.2–16.1 | |

[a]Wilcoxon matched-pairs signed-rank sum test. MNC, mononuclear cells.

**Table II** Median peak values (ranges) of haemopoietic progenitor cells per millilitre of peripheral blood

| | *Baseline* n=*11* | *Phase A Filgrastim 10 $\mu g\ kg^{-1}$* n=*11* | *Phase B Cyclophosphamide + Filgrastim 5 $\mu g\ kg^{-1}$* n=*11* | P-*value*[a] |
|---|---|---|---|---|
| MNC × $10^5\ ml^{-1}$ | 14<br>(2–28) | 145<br>(35–364) | 27<br>(12–169) | $P$<0.01 |
| CFU-GM $ml^{-1}$ | 53<br>(5–562) | 5504<br>(1850–12857) | 4915<br>(986–147 960) | $P$=0.9 |
| BFU-E $ml^{-1}$ | 108<br>(10–1043) | 4360<br>(1850–12 857) | 7776<br>(2242–109 069) | $P$=0.6 |
| $CD34^+ \times 10^3\ ml^{-1}$ | 2.7<br>(1.6–38) | 66<br>(17–548) | 75<br>(25–794) | $P$=0.6 |

[a]Wilcoxon matched-pairs signed-rank sum test between phase A and phase B.

600 mg $m^{-2}$ and carboplatin prescribed to an AUC 5 mg $ml^{-1}$ $min^{-1}$. Treatment is given 3 weekly for a total of six cycles.

The dose intensity for each drug was defined as the total amount of drug delivered per unit time, expressed as mg $m^{-2}$ $week^{-1}$ (Hryniuk, 1988) and relative dose intensity (Levin and Hryniuk, 1987) as the amount of drug delivered per unit time compared with the dose intensity of that drug in the standard single-drug regimen, i.e.

$$\frac{\text{Dose intensity in test regimen}}{\text{Dose intensity in standard regimen}}$$

For drug combinations the average relative dose intensity was calculated by dividing the sum of the relative dose intensities in the test regimen by the number of drugs in the regimen.

The six patients whose treatment was planned to be given at 3 weekly intervals had an intended average relative dose

**Figure 1** Cells mobilised into the peripheral blood during phase A and phase B.

intensity of 1.85 compared with our standard regimen, and the patients treated at 2 weekly intervals had an intended average relative dose intensity of 2.63. The first six patients treated at 3 weekly intervals received all cycles of chemotherapy as planned except for one patient who had a delay of 1 week after the third cycle of treatment owing to inadequate recovery of her platelet count. This resulted in the six patients treated at 3 weekly intervals receiving 96% of their planned chemotherapy on time and at full dose. The median average relative dose intensity actually delivered was 1.85; five of the six patients (83%) received the planned dose intensity.

We intended to treat seven patients at 2 weekly intervals but of these only two patients completed their treatment as planned. Two patients had a delay of 1 week, for one cycle and each of the remaining three patients had three delays of 1 week for each of the last three cycles. All the delays were due to inadequate recovery of the platelet count. The dose-limiting toxicity of the 2 weekly regimen was thrombocytopenia. In this cohort of patients only 61% of chemotherapy was delivered on time and at full dose. The median average relative dose intensity actually delivered for this group was 2.33 compared with an intended average relative dose intensity of 2.63; less than 30% of patients treated at 2 weekly intervals received their planned dose intensity (Table III).

In order to put the results of the dose-intensive arms of the study into context we have analysed a different group of ten patients treated with standard dose carboplatin (AUC 5 mg $ml^{-1}$ $min^{-1}$) and cyclophosphamide (600 mg $m^{-2}$) for six cycles, during the same period as those patients receiving dose-intensive treatment. Of these ten patients receiving standard treatment only two completed all planned cycles without any delay, i.e. 80% required at least one delay during treatment. Out of a total of 60 cycles of treatment, 22 cycles were delayed resulting in a median average relative dose intensity of 0.91 and only 56% of the planned average dose intensity delivered (Table III).

### *Neutropenic fever and haematological toxicity*

Five of the 14 patients developed neutropenic fever (defined as fever greater than 38°C and neutrophil count $<1.0\times10^9$ $l^{-1}$) requiring hospital admission and intravenous antibiotics using our standard policy. In four patients this occurred following cyclophosphamide 3 g $m^{-2}$ (phase B mobilisation). A further patient developed neutropenic fever during phase C treatment. There was no documented evidence of sepsis and all febrile patients had negative blood cultures. The one patient developing neutropenic fever during treatment phase C was being treated at 2 weekly intervals. All patients recovered following intravenous antibiotics and no patient required a delay in planned chemotherapy as a result of these episodes. All patients experienced grade 4 toxicity following cyclophosphamide 3 g $m^{-2}$ during phase B with nadir white blood counts $<1.0\times10^9$ $l^{-1}$ with corresponding absolute neutrophil counts $<0.5\times10^9$ $l^{-1}$. Table IV shows the median nadir white blood counts, absolute neutrophil and platelet counts and their ranges during the four cycles of carboplatin/cyclophosphamide (phase C) of treatment for the two groups of patients. The median WBC, ANC and platelet counts were generally higher for the patients treated at 3 weekly intervals compared with the equivalent cycle for the patients treated at 2 weekly intervals. No patient at any stage during the course of the study required the use of the reserve harvest from phase A.

### *Non-haematological toxicities*

One patient was withdrawn 3 days after entry owing to a skin reaction associated with filgrastim administered at

**Table IV** Median nadir white blood counts (WBC), absolute neutrophil counts (ANC) and platelet counts and their ranges during phase C

| Cycle number | Median nadir counts WBC × $10^9$ $l^{-1}$ (range) | ANC × $10^9$ $l^{-1}$ (range) | Platelet × $10^9$ $l^{-1}$ (range) |
|---|---|---|---|
| Three weekly treatment | | | |
| 2 | 1.9 (0.5–4.5) | 1.1 (0.3–3.0) | 74 (12–149) |
| 3 | 2.2 (1.0–4.5) | 0.9 (0.4–3.4) | 70 (32–255) |
| 4 | 2.0 (1.2–3.2) | 1.4 (0.4–2.2) | 64 (21–121) |
| 5 | 1.8 (0.7–4.2) | 1.9 (0.9–3.4) | 60 (14–130) |
| Two weekly treatment | | | |
| 2 | 1.3 (0.5–4.8) | 0.7 (0.1–3.7) | 142 (22–318) |
| 3 | 0.8 (0.3–3.5) | 0.4 (0.1–0.7) | 43 (21–173) |
| 4 | 1.1 (0.6–5.9) | 0.4 (0.1–3.7) | 29 (15–163) |
| 5 | 1.1 (0.6–6.3) | 0.4 (0.1–3.8) | 22 (9–80) |

**Table III** Planned dose intensity and delays in chemotherapy

| Regimen | Treatment interval (weeks) | Planned average dose intensity | Percentage of cycles delivered as planned | Median average dose intensity delivered (range) | Patients treated | Total number of Patients delayed | Cycles delivered | Cycles delayed |
|---|---|---|---|---|---|---|---|---|
| Cyclophosphamide 600 mg $m^{-2}$ Carboplatin AUC 5 mg $ml^{-1}$ $min^{-1}$ (six cycles) | 3 | 1 | 56 | 0.91 (0.78–1) | 10 | 8 | 60 | 22 |
| Cyclophosphamide 3 g $m^{-2}$ + Cyclophosphamide 900 mg $m^{-2}$ Carboplatin AUC 7.5 mg ml $min^{-1}$ (four cycles) | 3 | 1.85 | 96 | 1.85 (1.72–1.85) | 6 | 1[a] | 30 | 1 |
| Cyclophosphamide 3 g $m^{-2}$ + Cyclophosphamide 900 mg $m^{-2}$ Carboplatin AUC 7.5 mg $ml^{-1}$ $min^{-1}$ (four cycles) | 2[b] | 2.63 | 61 | 2.33 (2–2.63) | 7 | 5[a] | 35 | 11 |

[a]All the delays in treatment were due to inadequate recovery of platelets. [b]The treatment interval was 3 weeks after cyclophosphamide 3 g $m^{-2}$ and 2 weeks for carboplatin/cyclophosphamide.

10 $\mu$g kg$^{-1}$. This patient developed a disseminated maculopapular rash, with mild bone pain. All other patients successfully completed the planned administration of filgrastim, the only side-effect of note attributable to this drug being bone pain during recovery from the nadir blood count in phase A in one patient, and in three patients during phase C. No patient, whether treated at 2 or 3 weekly intervals, experienced more than grade 2 nausea or vomiting. Only one patient had grade 1 and one patient grade 2 vomiting. All patients experienced grade 2 alopecia. Two patients suffered grade 2 stomatitis and one patient grade 3. No patient suffered sensory loss during the study. Symptoms of constipation or diarrhoea occurred in nine patients but were only of grade 1 severity.

### Discussion

We have demonstrated that both filgrastim 10 $\mu$g kg$^{-1}$ given alone and cyclophosphamide 3 g m$^{-2}$ followed by filgrastim 5 $\mu$g kg$^{-1}$ result in effective mobilisation of peripheral blood progenitor cells in previously untreated patients with epithelial ovarian cancer. Although the median number of progenitor cells mobilised following cyclophosphamide 3 g m$^{-2}$ and filgrastim 5 $\mu$g kg$^{-1}$ was greater than that following mobilisation with filgrastim 10 $\mu$g kg$^{-1}$ alone, this difference was not statistically significant. There was considerable individual patient variation in the number of progenitor cells mobilised. Studies investigating the effects of filgrastim dose on mobilisation of progenitor cells have demonstrated that there is a dose–response relationship, with 10 $\mu$g kg$^{-1}$ of filgrastim mobilising more cells than 7.5 $\mu$g kg$^{-1}$, which in turn mobilises more than 5 $\mu$g kg$^{-1}$ (Stronceck *et al.*, 1994). In our study patients received filgrastim 10 $\mu$g kg$^{-1}$ alone during phase A but only 5 $\mu$g kg$^{-1}$ when used following cyclophosphamide (phase B), hence a possible explanation for the lack of significant difference between the two mobilisation regimens may be due to the difference in filgrastim dose administered. Our results are also very similar to those reported by Feremans *et al.* (1994) regarding the numbers of progenitor cells mobilised by G-CSF (10 $\mu$g kg$^{-1}$) alone compared with mobilisation produced using cyclophosphamide 4 g m$^{-2}$ and G-CSF 5 $\mu$g kg$^{-1}$ in the same patients being treated for myeloma. As we have not demonstrated a significant difference in progenitor cell mobilisation between the two regimens studied, it would be reasonable to use filgrastim alone (10 $\mu$g kg$^{-1}$) to mobilise normal healthy donors, thereby avoiding toxic chemotherapy in these patients. However, when mobilising patients with cancer it may be an advantage to incorporate chemotherapy as part of the mobilisation regimen, not only to enhance mobilisation of progenitor cells but also to provide effective treatment against the cancer and reduce the potential for reinfusion of viable malignant cells in the apheresis product.

Patients treated with the dose-intensive regimen at 3 weekly intervals tolerated the treatment extremely well and five of the six patients (83%) received the planned dose intensity of 1.85. Therefore, this 3 weekly intensive chemotherapy regimen of cyclophosphamide and carboplatin with filgrastim and progenitor cell support can be safely administered with very little haematological or systemic toxicity, while being able to deliver double the dose intensity (1.85:0.91) achieved in patients receiving our standard therapy.

Patients treated with the 2 weekly dose-intensive regimen suffered more delays compared with the 3 weekly dose-intensive regimen. Only two of the seven patients (29%) received their planned treatment on time and at full dose. Of the 35 cycles of chemotherapy delivered to this group of patients, 11 had to be delayed, all due to thrombocytopenia. We have reached the maximum tolerated dose by administering this dose-intensive regimen at 2 weekly intervals, with only 61% of planned cycles being delivered on time. Unless the platelet count can be supported further during the administration of such dose-intensive regimens it will not be possible to escalate the dose beyond 2 weekly therapy using this regimen. Although thrombocytopenia was dose limiting in the 2 weekly regimen, subjective toxicity and other haematological toxicities were no different from the 3 weekly therapy. The dose-intensive regimens described in the paper were equally as well tolerated as the standard regimen. However, increasing the dose intensity further would be less tolerable.

The overall response rate for the study patients was 70%, with 35% achieving complete remission, although care should be taken not to overinterpret these figures in view of the small numbers of patients involved.

Our study has demonstrated that 3 weekly dose-intensive chemotherapy can be administered safely with very low, and hence acceptable, levels of toxicity using peripheral blood progenitor cells and filgrastim support. However, escalating the dose intensity using a 2 weekly schedule resulted in less than a third of patients receiving their treatment as planned owing to thrombocytopenia. The 3 weekly dose-intensive schedule with double the dose intensity of our standard chemotherapy provides a regimen for evaluating the role of dose-intensive chemotherapy in patients with ovarian carcinoma within the context of a randomised trial with similar adverse effects in both treatment arms.

**Acknowledgements**
We would like to thank the Cancer Research Campaign for its generous support.

### References

ANDREWS R, BARTELMEZ S, KNITTER GH, MYERSON D, BERNSTEIN ID, APPELBAUM FR AND ZSEBO KM. (1992). A c-kit ligand, recombinant human stem cell factor, mediates reversible expansion of multiple CD34$^+$ colony-forming cell types in blood and marrow of baboons. *Blood*, **80**, 920–927.

BELLA M, COCCONI G, LOTTICI R, LEONARDI F, CECI G, PASSALACQUA R, DI BLASIO B, BORDI C, BOSCOTTINI B, MELPIGNANO M, DE BIASI D, FINARDI C AND BACCHI M. (1994). Mature results of a prospective randomised trial comparing two different dose intensive regimens of cisplatin in advanced ovarian carcinoma. *Ann. Oncol.*, **5**, (suppl.8), 2 (abstract).

CALVERT AH, NEWELL DR, GUMBRELL LA, O'REILLY S, BURNELL M, BOXALL FE, SIDDIK ZH, JUDSO JR, GORE ME AND WILTSHAW E. (1989). Carboplatin dosage; Prospective evaluation of a simple formula based on renal function. *J. Clin. Oncol.*, **7**, 11: 1748–1756.

CALVERT AH, LIND MJ, GHAZAL-ASWAD S, GUMBRELL L, MILLWARD MJ, BAILEY NP, DORE-GREEN F, CHAPMAN F, SIMMONS D AND PROCTER M. (1994). Carboplatin and granulocyte colony-stimulating factor as first line treatment for epithelial ovarian cancer: A phase I dose-intensity escalation study. *Semin. Oncol.*, **21**, (suppl.12), 1–6.

COLOMBO N, PITTELLI MR, PARMA G, MARZOLA M, TORRI W AND MANGIONI C. (1993). Cisplatin (P) dose intensity in advanced ovarian cancer (AOC): a randomised study of conventional dose (DC) *vs* dose-intensive (DI) cisplatin monochemotherapy. *Proc. Am. Soc. Clin. Oncol.*, **12**, 255.

DUHRSEN U, VILLEVAL J-L, BOYD J, MORSTYN G AND METCALF D. (1988). Effects of recombinant human granulocyte colony-stimulating factor on hemopoietic cells in cancer patients. *Blood*, **72**, 2074–2079.

FEREMANS W, LE-MOINE F, RAVOET C, LAMBERMONT M, BASTIN G, DELVILLE JP, PRADIER O, DUPONT E AND CAPEL P. (1994). Optimal blood stem cell mobilisation using 10 micrograms/kg granulocyte colony-stimulating factor (G-CSF) alone for high-dose melphalan intensification in multiple myeloma: an intrapatient controlled study. *Am. J. Hematol.*, **47** (2), 135–138.

GIANNI AM, SIENA S, BREGNI M, TORELLA C, STERN AC, PILERI A AND BONNADONNA G. (1989). Granulocyte-macrophage colony-stimulating factor to harvest circulating haemopoietic stem cells for autotransplantation. *Lancet*, **2**, 580.

HRYNIUK W. (1988). The importance of dose intensity in outcome of chemotherapy. In *Important Advances in Oncology*. Hellman S and Rosenberg S. (eds). pp. 121–141. Lippincott: Philadelphia, PA.

JUTTNER CA, TO LB, DYSON PG, HAYLOCK DN AND ROBERTS MM. (1992). Comparison of haematological recovery, toxicity and supportive care of autologous PBPC, autologous BM and allogeneic BM transplants. *Int. J. Cell Cloning*, **10**, 160–164.

KAYE S, LEWIS C, PAUL J, DUNCAN ID, GORDAN HK, KITCHENER HC, CRUICKSHANK DJ, ATKINSON RJ, SOUKOP M AND RANKIN EM. (1992). Randomised study of two doses of cisplatin with cyclophosphamide in epithelial ovarian carcinoma. *Lancet*, **340**, 329–333.

LEVIN L AND HRYNIUK W. (1987). Dose intensity analysis of chemotherapy regimens in ovarian carcinoma. *J. Clin. Oncol.*, **5**, 756–767.

MCGUIRE WP, HOSKINS WJ, BRADY MF, HOMESLEY HD, CLAKE-PEARSON DL. (1992). A phase III trial of dose intense versus standard dose cisplatin and cytoxan in advanced ovarian cancer. *Proc. Am. Soc. Clin. Oncol.*, **11**, 226.

MURPHY D, CROWTHER D, RENNISON J, PRENDIVILLE J, RANSON M, LIND M, PATEL U, DOUGAL M, BUCKLEY CH AND TINDALL VR. (1993). A randomised dose intensity study in ovarian carcinoma comparing chemotherapy given at four week intervals for six cycles with half dose chemotherapy given for twelve cycles. *Ann. Oncol.*, **4**, 377–383.

NGAN HYS, CHOO YC, CHEUNG M, WONG LC, MA HK, COLLINS R, FUNG C, NG CS, WONG V AND HO HC. (1989). Hong Kong Ovarian Carcinoma study group. A randomised study of high dose versus low dose cisplatin combined with cyclophosphamide in the treatment of advanced ovarian cancer. *Chemotherapy*, **35**, 221–227.

PARKIN DM, LAARA E AND MUIR CS. (1988). Estimates of the worldwide frequency of sixteen major cancers in 1980. *Int. J. Cancer*, **41**, 184–197.

RUSSELL NH, HUNTER A, ROGERS S, HANLEY J AND ANDERSON D. (1993). Peripheral blood stem cells as an alternative to marrow for allogeneic transplantation. *Lancet*, **341**, 1482.

SIENA S, BREGNI M, BONSI L, SKLENAR I, BAGNARA GP, BONNADONNA G AND GIANNI GM. (1991). Flow cytometry for clinical estimation of circulating hematopoietic progenitors for autologous transplantation in cancer patients. *Blood*, **77**, 400–409.

SOCINSKI MA, ELIAS A, SCHNIPPER L, CANNISTRA SA, ANTMAN KH AND GRIFFIN JD. (1988). Granulocyte–macrophage colony-stimulating factor expands the circulating haemopoietic progenitor cell compartment in man. *Lancet*, **1**, 1194–1196.

STRONCECK D, CLAY M, JASZCZ W, MILLS B, OLDHAM F AND MCCULLOUGH J. (1994). Longer than 5 days G-CSF mobilisation of normal individuals results in lower $CD34^+$ cell counts. *Blood*, **84**, (suppl.1), 2149.